# “Off-Spotter”: very fast and exhaustive enumeration of genomic lookalikes for designing CRISPR/*Cas* guide RNAs

**DOI:** 10.1186/s13062-015-0035-z

**Published:** 2015-01-29

**Authors:** Venetia Pliatsika, Isidore Rigoutsos

**Affiliations:** Computational Medicine Center, Jefferson Alumni Hall, #M81, Sidney Kimmel Medical College at Thomas Jefferson University, 1020 Locust Street, Philadelphia, PA 19107 USA

**Keywords:** CRISPR, Off-targets, Cas endonucleases, Cas9, Guide RNAs, gRNAs, Indexing, Hashing

## Abstract

**Background:**

CRISPR/Cas (Clustered Regularly Interspaced Short Palindromic Repeats/CRISPR associated nucleases) is a powerful component of the prokaryotic immune system that has been adapted for targeted genetic engineering in higher organisms. A key element of CRISPR/Cas is the “guide” RNA (gRNA) that is ~20 nucleotides (nts) in length and designed to be complementary to the intended target site. An integral requirement of the CRISPR/Cas system is that the target site be followed by a protospacer adjacent motif (PAM). Care needs to be exercised during gRNA design to avoid unintended (“off-target”) interactions.

**Results:**

We designed and implemented the Off-Spotter algorithm to assist with the design of optimal gRNAs. When presented with a candidate gRNA sequence and a PAM, Off-Spotter quickly and exhaustively identifies all genomic sites that satisfy the PAM constraint and are identical or nearly-identical to the provided gRNA. Off-Spotter achieves its extreme performance through purely algorithmic means and not through hardware accelerators such as graphical processing units (GPUs). Off-Spotter also allows the user to identify on-the-fly how many and which nucleotides of the gRNA comprise the “seed”. Off-Spotter’s output includes a histogram showing the number of potential off-targets as a function of the number of mismatches. The output also includes for each potential off-target the site’s genomic location, a human genome browser hyperlink to the corresponding location, genomic annotation in the vicinity of the off-target, GC content, etc.

**Conclusion:**

Off-Spotter is very fast and flexible and can help in the design of optimal gRNAs by providing several PAM choices, a run-time definition of the seed and of the allowed number of mismatches, and a flexible output interface that allows sorting of the results, optional viewing/hiding of columns, etc. A key element of Off-Spotter is that it does not have a rigid definition of the seed: instead, the user can declare both the seed’s location and extent on-the-fly. We expect that this flexibility in combination with Off-Spotter’s speed and richly annotated output will enable experimenters to interactively and quickly explore different scenarios and gRNA possibilities.

**Reviewed:**

This article was reviewed by Dr Eugene Koonin and Dr Frank Eisenhaber.

**Electronic supplementary material:**

The online version of this article (doi:10.1186/s13062-015-0035-z) contains supplementary material, which is available to authorized users.

## Implementation

### Introduction

Many prokaryotes have evolved a natural defense mechanism against plasmids [1] and viruses [2,3] that is known as the CRISPR/Cas nuclease system. The system has been found by now in many archaeal and bacterial organisms and is part of their immune response that effectively implements an acquired resistance mechanism to viral infections.

**Figure 1:**
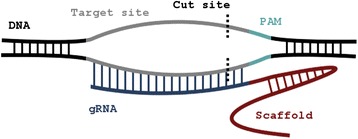
**A CRISPR-Cas site.**

There are two components to the CRISPR/Cas system [4-6]. The first component is represented by the CRISPR loci. These loci comprise non-contiguous repeats that are interrupted by short sequence segments known as “spacers.” The sequence composition of each spacer is variable and matches the DNA sequence of elements foreign to the prokaryotic genome at hand such as phages and plasmids. The second component is represented by the Cas nuclease genes that are generally proximal to the CRISPR loci. The CRISPR loci are transcribed and processed into short RNAs that, in turn, guide the *Cas* nucleases to the foreign DNA sites to be cleaved, see Figure 1. Thanks to its conceptual simplicity and power the system was quickly adapted for many uses in the cells of higher organisms [7-12].

To experimentally implement the CRISPR/Cas system for a specific goal one needs to design a “guide” sequence, the gRNA, that is typically 20 nts in length and complementary to the intended target [13-16]. An additional requirement is that the targeted sequence be followed by a PAM sequence such as NGG, NAG, NNNNACA, etc. [17]. Note that we use N to refer to any of the four nucleotides, i.e. N stands for any of A, C, G or T.

Since the underlying system is so powerful, the ability to target precisely and to minimize unintended interactions, i.e. the “off-targets”, is of paramount importance. Intimately linked to the concept of the “off-targets” is the concept of the gRNA “seed”, which is defined as that segment of nucleotides of the gRNA that is necessary before the interaction of the gRNA with its target can occur. The gRNA seed appears to be analogous to the miRNA seed [18-21] and is currently believed to span between five and eight nucleotides counting from the 3′ end of the gRNA; the details of what constitutes a seed in this context are currently a matter of active research [7,13,15,16,22-28].

As we have shown previously, there are many DNA sequences that have numerous genomic instances and are also present in mRNAs [29,30], intronic space [31], or in the vicinity of transcription start sites [32]. The copy numbers of these DNA sequences can be very high even in the presence of the PAM constraint; it is therefore important that an off-target discovery tool be able to very quickly enumerate and report all genomic hits, especially if the query belongs to one of these special cases.

Several solutions have been proposed to date for tackling the off-target discovery problem [9,33-37]. These solutions can be either slow or not exhaustive. Additionally, they are not flexible in that their definition of the “seed” is typically hardwired and part of the design of the method. As such, the user lacks the ability to define on-the-fly the seed’s location and/or span.

### Problem definition

The more general version of the problem has as follows: “given a potentially large genome sequence *G* composed of four nucleotides (A, C, G, T), a query sequence *Q*, a PAM string, and a number *M*, identify and report all locations in *G* where the underlying sequence *q* is exactly the same length as *Q*, has *M* or fewer mismatches when compared to *Q* and is immediately followed by the given PAM.” In a more constrained version of the problem, any claimed off-targets *q* must also satisfy a “seed” constraint whereby any look-alike sequence *q* and the query *Q* must contain identical nucleotides in all of the positions that comprise the seed. For realistic instances of the problem, *G* is in the order of billions of nucleotides, *Q* = 20, and *M* ≤ 5.

### Our solution

**Figure 2:**
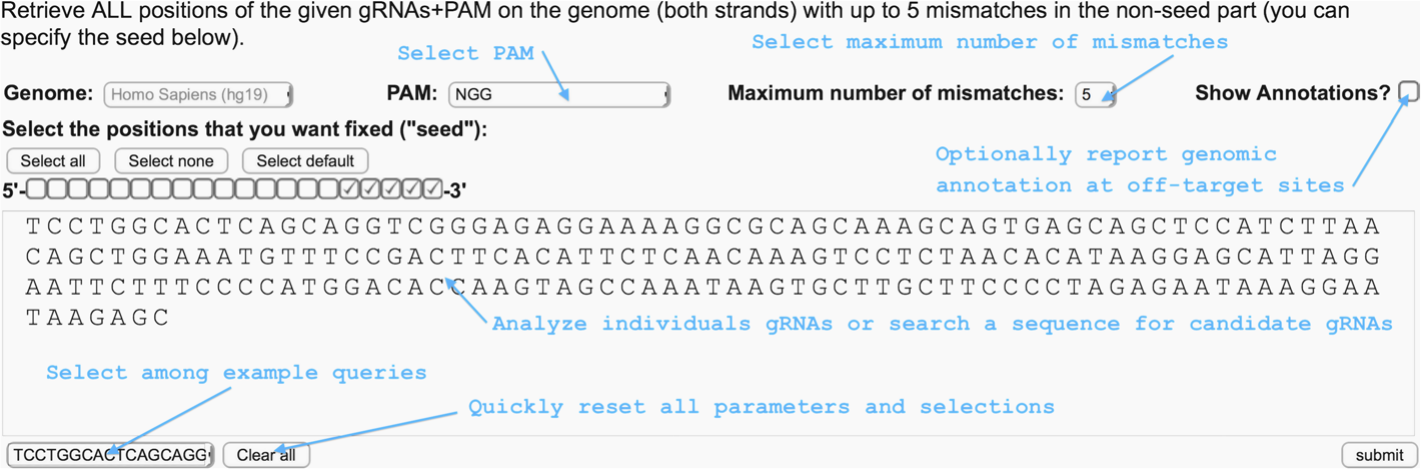
**Entry form of Off-Spotter.** The user can enter one or multiple gRNAs or a target sequence for analysis. The user can also select among 4 PAM options, the maximum number of mismatches, annotation display and seed size and location.

**Figure 3:**
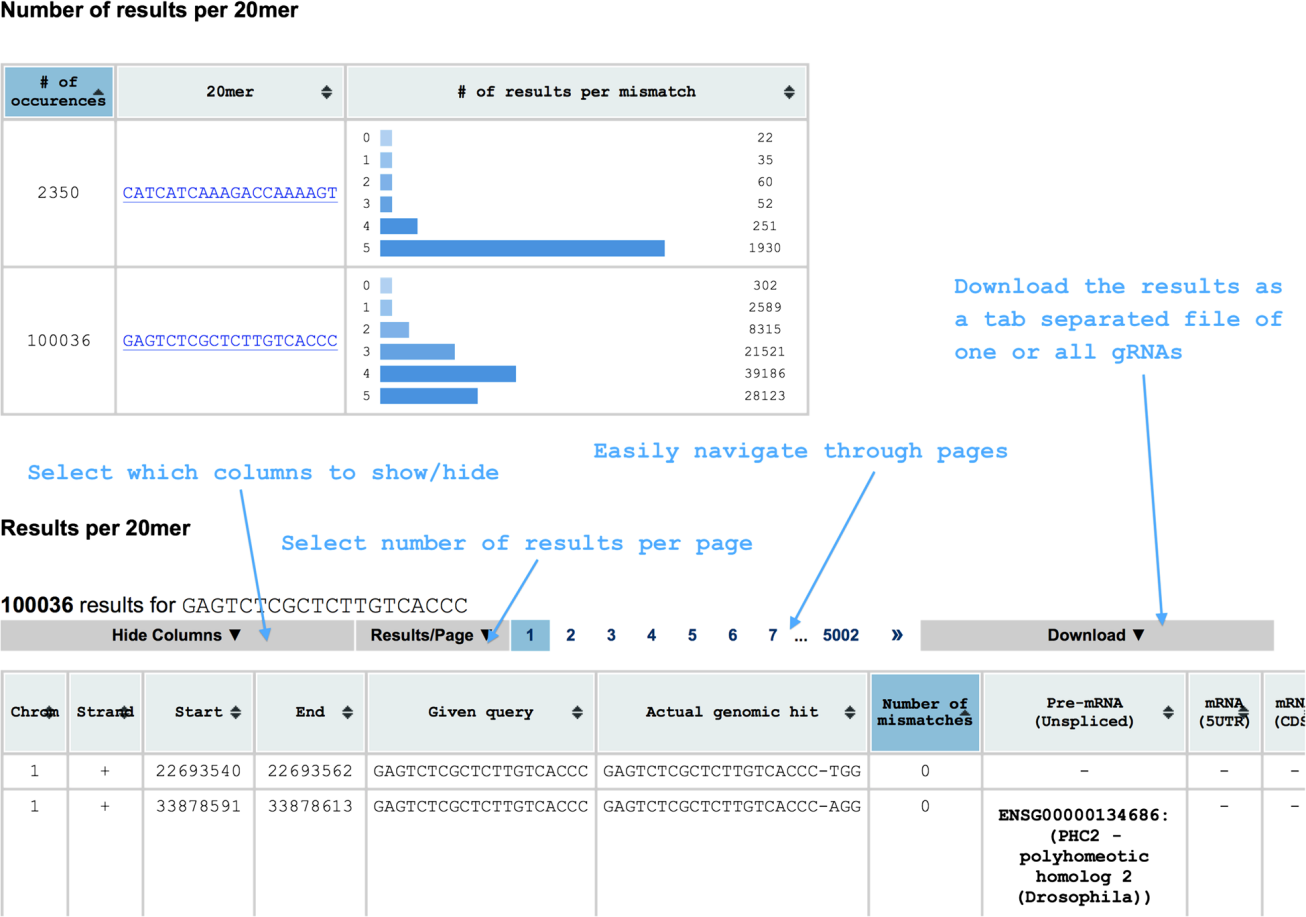
**Output page of Off-Spotter.** As seen in the figure, the user can sort the results by all the information presented in the columns and also by multiple columns at the same time, can show/hide columns, download the results, select the number of results presented per page and navigate through the pages easily. The mismatches are indicated by red lowercase letters for each off-target. The annotation reports include the ENSEMBL gene identifiers, transcript identifiers, and common gene names. Two types of tables are available per search. The top table provides the summary of the results, i.e. the number of results per gRNA found in the input, the strand on which it was found in those instances where a target sequence was entered, and a histogram of the number of off-targets as a function of the number of mismatches. The table for each individual gRNA includes all off-targets for the corresponding gRNA together with detailed genomic location information and genomic annotation. See also text.

We have developed “Off-Spotter”, a very fast and exhaustive algorithmic solution to the off-target discovery problem. Our implementation is available for interactive use through https://cm.jefferson.edu/Off-Spotter/. For each search, we provide users with the option to use checkmark buttons to define how many and which nucleotides of the query *Q* comprise the seed and, thus, must be identically present in all off-target sites to be reported, to select a PAM sequence to use, the maximum desired number of mismatches, and whether to report genomic annotation information at the off-target sites (Figure 2). For each potential off-target site that Off-Spotter reports (Figure 3), we list the site’s chromosome id, strand id, location within the chromosome, the corresponding genomic sequence *q* with the mismatches between *q* and *Q* indicated by red lower case letters, the actual number of mismatches between *q* and *Q*, the gene and transcript information of each site, and, optionally, whether the off-target is located in a 5′ untranslated region (5′UTR), an amino acid coding sequence (CDS), a 3′UTR, a long non-coding RNA (lincRNA), etc. Additionally, we report each off-target’s GC content and provide a hyperlink to the UCSC genome browser. For each off-target we also report the fully dereferenced instance of the PAM for PAM strings (e.g. **N**GG, **N**AG, **NNNN**ACA) with unspecified nucleotides. Separately for each analyzed gRNA, we also report a histogram of the number of potential off-targets as a function of the mismatches that they have when compared to the gRNA sequence.

### The algorithm

Off-Spotter comprises a one-time “off-line” stage during which two tables holding the pre-computed results are built, and an “on-line” stage during which queries are serviced and the list of potential off-targets is returned. Following the retrieval of the off-targets, during an *optional* third stage, Off-Spotter attaches annotation information to the off-targets. In order to embark on the first stage of Off-Spotter, we need to know each PAM sequence that will ever be used. Our current implementation incorporates several PAMs that range in length from three (3) to seven (7) nucleotides. Additional PAMs can be incorporated trivially.

#### The algorithm: the 1st stage (“off-line")

**Figure 4:**
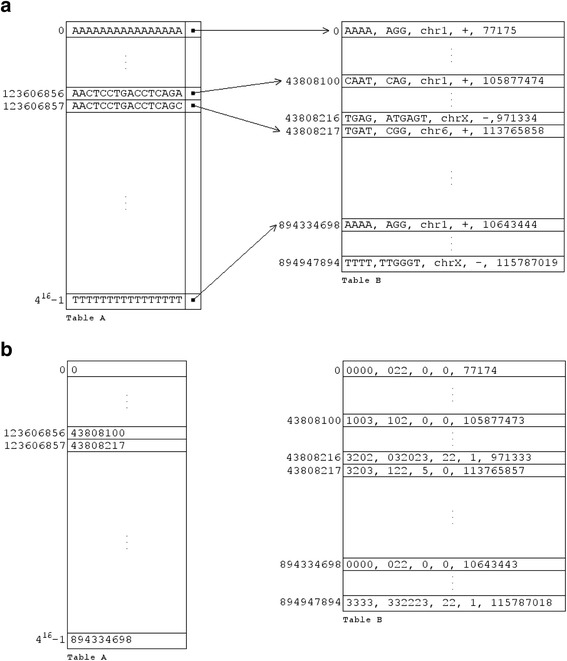
**Data structures used by Off-Spotter.** A schematic of Tables A and B that are used by Off-Spotter with example entries. The tables are created during the “offline” stage of the algorithm. **a)** Table A here is the hash table that contains all possible 16-mers and a pointer to table B for each 16-mer. Table B contains the information of each hit, i.e. the last 4 bases of the gRNA, PAM, chromosome, strand, and starting position within the chromosome. **b)** the Tables in this panel show the same entries and can be interpreted as follows: each of the last 4 bases of the gRNAs and the PAM get assigned a number according to the scheme A → 0, C → 1, G → 2, T → 3 chromosomes get assigned a number from 0 to 24 (chr 1 → 0, chr 2 → 1, … chr Y → 23, MT → 24); strands are represented by 0 (forward) or 1 (reverse); and, all positions are 0-based.

During this stage we build the two tables A and B that will be used during execution (Figure 4). Table A is 1-dimensional and contains 4^16^ cells; each cell corresponds to a different 16-mer composed of the four nucleotides A, C, G and T. Assuming that A → 0, C → 1, G → 2 and T → 3, we can map any string of 16 nucleotides, e.g. AACTCCTGACCTCAGA, to a number, in this case 0013113201131020, and then to a unique cell *i* of Table A using the following hash function: *i* = 0*4^15^ + 0*4^14^ + 1*4^13^ + 3*4^12^ + 1*4^11^ + 1*4^10^ + 3*4^9^ + 2*4^8^ + 0*4^7^ + 1*4^6^ + 1*4^5^ + 3*4^4^ + 1*4^3^ + 0*4^2^ + 2*4^1^ + 0*4^0^ = 123,606,856. The values of *i* can range from 0 to 4^16^-1 = 4,294,967,295 inclusive and there is exactly one value *i* for each string of 16 nucleotides, and *vice versa*. For the example at hand, cell *i* in Table A contains a pointer to a cell *i´* of Table B where we include information about *all* the genomic locations where we can find the 16-mer AACTCCTGACCTCAGA followed by any four nucleotides, followed by PAM. We use **PAM** in boldface as part of a nucleotide string to denote any of the oligonucleotide PAMs that are considered. Tables A and B effectively form groups out of all 20-mer strings that are present in the genome and are followed by PAM by using the first 16 nucleotides of each such 20-mer. It is easy to determine how many 20-mer strings exist that look like AACTCCTGACCTCAGA-NNNN-**PAM**. Indeed, note that the 16-mer AACTCCTGACCTCAGA of our example string maps to a cell *i* of Table A that in turn points to location *i´* of Table B. Note now that the string AACTCCTGACCTCAG**C**, which differs from AACTCCTGACCTCAG**A** in its last letter, will map to cell *j* = *i + 1* of Table A that points to location *j´* of Table B. As can be seen from Figure 4A, the difference *j´*-*i´* is exactly equal to the number of distinct 20-mers AACTCCTGACCTCAGA-NNNN that are present in the genome and are followed by all PAM strings that are used by the implementation of the algorithm, i.e., all the 20-mers that are flanked on the right by any of the considered PAM strings and share the same first 16 letters, in this case AACTCCTGACCTCAGA.

To populate the two Tables A and B we scan the genome twice (we process both strands of the genome during a scan). During the first scan, we enumerate all genomic 20-mers N_1_N_2_N_3_…N_15_N_16_(N_17_N_18_N_19_N_20_)-**PAM** – recall that N is any of A, C, G or T – and count how many of these 20-mers start with the same 16-nucleotide string N_1_N_2_N_3_…N_15_N_16_ in order to allocate adequate space for Table B and properly initialize all pointers from Table A to Table B. Note that the number of entries in Table B is at most equal to the number of locations in the genome where one can find any of the used PAM strings. During the second scan, we populate Table B by storing in it the genomic locations of all N_1_N_2_N_3_…N_15_N_16_(N_17_N_18_N_19_N_20_)-**PAM** as well as the actual nucleotide sequence N_17_N_18_N_19_N_20_ and the PAM at each such location. Note that we do not need to store N_1_N_2_N_3_…N_15_N_16_ as it can be recovered uniquely by inverting the above polynomial computation.

In order to make optimal use of storage and minimize memory requirements, for each N_1_N_2_N_3_…N_15_N_16_(N_17_N_18_N_19_N_20_)-**PAM** we use a bit vector to represent each of: the string N_17_N_18_N_19_N_20_, the chromosome id, the strand id, the chromosome location, and the actual PAM string. This allows us to use more than one PAMs in the same data structure. We require 8 bits for N_17_N_18_N_19_N_20_ (2 bits per nucleotide/A → 0, C → 1, G → 2 and T → 3), 3 bits for a PAM id (it allows up to 2^3^ = 8 distinct PAMs – clearly it can be increased if one needs to accommodate more PAMs), 2 bits for each unspecified nucleotide of a used PAM, 5 bits for the chromosome id (we need to represent the 25 values that correspond to chromosomes 1 through 22, X, Y and MT), 1 bit for the strand id (forward or reverse), and 28 bits for the position within a chromosome (we must be able to accommodate chromosome 1, which is the longest at more than 250 million bases) – see also Figure 4B.

#### The algorithm: the 2nd stage (“on-line”)

When presented with a query *Q* = N_1_N_2_N_3_…N_15_N_16_(N_17_N_18_N_19_N_20_) and a PAM, we first enumerate all 20-mers N´_1_ N´_2_ N´_3_…N´_15_ N´_16_(N´_17_ N´_18_ N´_19_ N´_20_) that differ from *Q* at fewer than *M* or fewer positions, then use the 16-mer N´_1_ N´_2_ N´_3_…N´_15_ N´_16_ to find its location in Table A, and, follow the corresponding pointer to Table B. As there exist at most $$ {\displaystyle {\sum}_{i=0}^M\kern0.5em \left(\begin{array}{cc}\hfill \left(\begin{array}{c}\hfill 20\hfill \\ {}\hfill i\hfill \end{array}\right)\hfill & \hfill {3}^i\hfill \end{array}\right)} $$ possible 20-mers that can differ at *M* or fewer positions from *Q* we will never follow more than that many pointers from Table A to Table B. E.g. for *M* = 5, we will never follow more than 4,192,468 pointers: even though this may seem like a large number, these operations are very fast to execute, as we will see below, and lend Off-Spotter its speed. For every pointer contained in a cell *i* of Table A we recover a location *i*´ of Table B. Then we reconstitute all of the strings N´_1_ N´_2_ N´_3_…N´_15_ N´_16_(N´´_17_ N´´_18_ N´´_19_ N´´_20_)-**PAM**´´ using the entries N´´_17_ N´´_18_ N´´_19_ N´´_20_-**PAM**´´ of Table B that start at position *i*´ and for which the user-selected PAM string matches PAM´´. Therefore, if the reconstituted 20-mer N´_1_ N´_2_ N´_3_…N´_15_ N´_16_ (N´´_17_ N´´_18_ N´´_19_ N´´_20_) differs from the user-provided query N_1_N_2_N_3_…N_15_N_16_(N_17_N_18_N_19_N_20_) at *M* or fewer locations and these locations also satisfy the user-provided constraints regarding the location and span of the seed then Off-Spotter reports the 20-mer as a potential off-target.

#### The algorithm: the annotation stage (optional)

To increase the usefulness of Off-Spotter, we also make available ENSEMBL [38] annotations for each reported off-target. For each off-target that has been retrieved during the previous stage, we use its coordinates to search the ENSEMBL annotation files for potential overlaps; when an overlap is found, the corresponding ENSEMBL gene and transcript identifier are returned. For performance purposes, we presort the annotation files and index them by chromosome id. The annotation step is logically distinct from the identification of off-targets and we provide it as a user option. Even though tagging the off-targets with genomic annotation adds to the length of the run, the running time is still extremely fast. The running times that the user experiences when annotation is enabled are still measured in seconds and shown in Additional file 1.

### Results

We built the current implementation of Off-Spotter using the hg19 human genome assembly and allow the user to select among four different PAMs, define the maximum number of mismatches (up to 5), decide whether to report annotations, and delineate the seed’s location and extent on-the-fly. The user can enter up to twenty 20-mers (20-mers need to be separated by new lines) or a genomic sequence of interest (up to 500 nucleotides). If one or more 20mers are entered, each 20-mer is processed in turn and the off-target results reported on separate tables, one table per 20-mer. If a sequence is given, Off-Spotter will scan the forward and reverse strand of the sequence automatically to identify all 20-mers N_1_N_2_N_3_…N_15_N_16_N_17_N_18_N_19_N_20_ that are followed by the user-selected PAM: each such 20-mer will be processed separately. The user can sort the reported potential off-targets separately for each 20-mer by chromosome id, strand id, number of mismatches, and actual off-target sequence. Additionally, off-targets can be sorted based on whether they overlap 5′UTR, CDS, 3′UTR, unspliced mRNAs, unspliced lincRNAs, spliced lincRNAs, or off-target GC-content. The user can also sort the reported off-targets by two or more of these fields simultaneously. To facilitate the users’ navigation through the reported output, each column can be hidden/un-hidden at will. Finally, the user can download the generated results as tab-separated files either for select 20-mers or for all analyzed 20-mers together. In all instances we also provide for each potential off-target a hyperlink to the UCSC genome browser to enable visualization of the corresponding off-target site in its genomic context. Our short-term plans include extending Off-Spotter’s engine to enable analyses of the mouse genome, and providing more PAM choices. As more information becomes available on rules to use to rank gRNAs we will be enhancing Off-Spotter accordingly.

### Run-time performance

As we have mentioned, one of Off-Spotter’s key attributes is its performance. In fact, even though Off-Spotter does *not* make use of GPUs it can nonetheless achieve very short run-times by software means alone. In Table 1, we report the run-times for several queries that are meant to span the gamut of off-target results that a query can generate. The run-times correspond to running Off-Spotter as a single-thread process on a 64-bit system powered by an Intel Xeon with a clock speed of 2.66 GHz. As can be seen, a typical query requires only a fraction of a second to complete. Even pathological queries that necessitate the generation and reporting of hundreds of thousands of potential off-targets are completed in a few seconds.

**Table 1:** **Run times for 20-mer queries**

**QUERY**	**PAM**	**Time (in seconds)**	**Number of retrieved potential off-targets (≤5 mismatches)**
ATTCGCGGCAAAGGAGGAGA	NNGRRT	0.723	259
ATTCGCGGCAAAGGAGGAGA	NNNNACA	0.718	326
AACTCCTGACCTCAGCAAAA	NNGRRT	0.773	677
CATCATCAAAGACCAAAAGT	NNGRRT	0.801	705
AACTCCTGACCTCAGAAAAA	NNGRRT	0.768	894
ATTCGCGGCAAAGGAGGAGA	NGG	0.729	1,223
CATCATCAAAGACCAAAAGT	NNNNACA	0.765	1,485
ATTCGCGGCAAAGGAGGAGA	NAG	0.735	1,554
AACTCCTGACCTCAGCAAAA	NNNNACA	0.775	2,077
CATCATCAAAGACCAAAAGT	NGG	0.802	2,350
AACTCCTGACCTCAGAAAAA	NNNNACA	0.790	2,407
AACTCCTGACCTCAGCAAAA	NGG	0.778	3,612
AACTCCTGACCTCAGAAAAA	NGG	0.773	4,087
CATCATCAAAGACCAAAAGT	NAG	0.769	4,947
AACTCCTGACCTCAGCAAAA	NAG	0.791	5,406
CTTTGGGAGGCTGAGGTGGG	NNNNACA	0.986	5,907
AACTCCTGACCTCAGAAAAA	NAG	0.813	7,334
CTTTGGGAGGCTGAGGTGGG	NNGRRT	0.898	10,347
AAAAAAAAAAAAAAAAAAAA	NNGRRT	2.401	220,857
CTTTGGGAGGCTGAGGTGGG	NAG	1.682	241,707
TTTTTTTTTTTTTTTTTTTT	NNNNACA	2.945	458,847
TTTTTTTTTTTTTTTTTTTT	NGG	3.172	549,466
TTTTTTTTTTTTTTTTTTTT	NNGRRT	3.991	554,972
AAAAAAAAAAAAAAAAAAAA	NNNNACA	3.637	614,732
AAAAAAAAAAAAAAAAAAAA	NGG	3.320	617,790
CTTTGGGAGGCTGAGGTGGG	NGG	3.828	708,125
TTTTTTTTTTTTTTTTTTTT	NAG	15.143	1,807,996
AAAAAAAAAAAAAAAAAAAA	NAG	12.092	1,902,731

The chosen 20-mers are meant to sample the whole gamut of potential off-targets that a candidate gRNA may generate**.** The entries also demonstrate how impactful the chosen PAM can be on the number of potential off-targets. The times shown do not include annotation reports. We note here that the number of results of AACTCCTGACCTCAGAAAAA shown in this Table are not expected to agree with the number of results for AACTCCTGACCTCAGAAAAA that are shown in Figure 4: this Table shows all potential targets of AACTCCTGACCTCAGAAAAA for a specific PAM with up to 5 mismatches and an unspecified seed whereas Figure 4 shows the number of all 20-mers that have the form AACTCCTGACCTCAGANNNN, where N = {A,C,G,T} and are followed by any of the PAMs that we have implemented.

### Comparison with other tools

In Additional file 1 and Additional file 2, we present comparisons with other currently available tools. Additional file 1 presents a quantitative comparison of the various tools based on their run-time and the number of off-targets that they report. Additional file 2 provides a summary of the features that characterize the various tools.

### Discussion

We have described Off-Spotter, our algorithmic solution to the problem of determining potential off-targets for candidate gRNAs. Off-Spotter guarantees that it will retrieve and report all genomic locations that differ by a user-specified number of positions from the query gRNA and which also satisfy a user-specified seed location/extent constraint: the user can make both choices prior to each run.

Naturally, whether the reported genomic loci will correspond to *bona fide* off-targets depends on other criteria that can include the chosen span of the seed (5 nts, 6nts, 7nts, etc.), the extent of similarity between the off-target and the gRNA in the region immediately beyond the seed, chromatin accessibility information, methylation status, and other considerations [27]. It is worth noting that attributes such as chromatin accessibility and methylation status do not exist for all cell types. Moreover, such attributes are expected to differ across cell types. Nonetheless, when available, this information can be easily taken into account simply by post-processing the output generated by Off-Spotter.

### Conclusions

Off-Spotter is a system that identifies and reports all potential off-target sites for a given gRNA and PAM combination. Off-Spotter is very fast and provides a richly annotated output while also enabling the user to interact with the generated results. We expect that Off-Spotter will be a great addition to the collection of tools that are available to researchers who want to harness the power of the CRISPR/Cas system.

## Availability and Requirements

**Project name:** Off-Spotter

**Project home page:**https://cm.jefferson.edu/Off-Spotter/

**Operating system(s):** RedHat Linux 6.6

**Programming language:** C/C++

**Other requirements:** a minimum of 32 GB of RAM

**License:** No restrictions for research, academic, and other not-for-profit activities. For complete list of terms see https://cm.jefferson.edu/downloads/Off-Spotter-code/

**Any restrictions to use by non-academics:** license needed

## Reviewers comments

### Reviewer 1: Dr. Eugene Koonin

Off-Spotter described by Pliatsika and Rigoutsos is new software that addresses the paramount problem in the CRISPR-mediated genome engineering, namely identification of off-target sites that might be recognized by a given guide RNA.

The software appears to be very well designed and even includes the reverse procedure, namely identification of all potential guide RNA recognition sites preceded by a PAM in any nucleotide sequence. As the authors point out themselves, this is a relatively crude, straightforward tool that only enumerates potential off-target recognition sites but does not provide information on the actual efficacy of their recognition. However, I would not realistically call this a drawback because with the current state of the art, a truly accurate method for predicting offtarget sites does not seem to be attainable.

I believe that Off-Spotter will be an extremely useful tool as is and will be in high demand as soon as it is made available. Again, as the authors note, there is excellent potential for further improvements.

Quality of written English: Acceptable

Response: *We thank the Reviewer for his positive comments. The Reviewer is indeed correct that it is not realistic at this point in time to expect a computational tool to accurately enumerate only ‘true’ off-targets while discarding ‘false positives’. It is possible however, and we believe important and useful to the field’s practitioners, for such computational tools to provide an exact count of the potential number of off-targets, an “upper bound” of sorts, that a candidate guide RNA can have: this is precisely the question that Off-Spotter aims to address in this first iteration.*

### Reviewer 2: Dr Frank Eisenhaber

The tool “off-spotter” relies on a possibly incompletely understood description of the genomic motif recognized by the CRISPR/Cas system and has the goal to enumerate all possible genomic hits for given gRNA and PAM sequences. Since this is a computationally challenging task, the authors venture for a GPU-based software solution and offer a WWW server access.

It appears to the reviewer that the area is evolving fast due to general demand and there is quite some literature out already. Unfortunately, the authors do not elaborate much about alternative projects in the field and, therefore for the reader, it is difficult to fully assess the progress in this work. In addition to the 3 references mentioned, there are also CRISPRseek (PLoS One. 2014 Sep 23; 9(9):e108424), CHOPCHOP (Nucleic Acids Res. 2014 Jul; 42(Web Server issue): W401-7), etc. A recent review about rational design has appeared (Nat Biotechnol. 2014 Sep 3. doi:10.1038/nbt.3026).

Naively, it appears that the task resolved by Off-Spotter is just one element of the problem that needs to be solved in the applied setting. Maybe, the first gRNA is selected not very well and one might be tempted to ask whether the program can suggest a better alternative instead of torturing Off-Spotter with more manually selected gRNA examples. How does the competition fare in this respect and can this functionality be put on top of the existing Off-Spotter?

Since the typical reader might not be very familiar with all the molecular biology detail, it might be helpful to add a figure that shows the principal structure of a CRISPR/Cas site in the genome together with a typical example sequence.

For wider usage of the tool, the authors are strongly advised to make their tool’s software (best the source code together some compiled versions) available for download.

As minor note, it is advised to add the full translation of the abbreviation “CRISPR/Cas” in the abstract.

There is no doubt that a fast, GPU-based solution for the enumeration problem is useful and desired by the community.

Quality of written English: Acceptable

Response: *We thank the Reviewer for his comments. We found the Reviewer’s suggestions very useful and have addressed them all in the revised version of our manuscript. Specifically, we have incorporated the following additions and changes:*

- *We added references to a few more tools including the two mentioned by the Reviewer.*
- *We carried out a comprehensive comparison of Off-Spotter and other available tools. There are two aspects to this comparison. First, we compared the various tools from the standpoint of their speed and ability to enumerate exhaustively and quickly all potential off-targets that a candidate guide RNA could have. Second, we compared the various tools from the standpoint of their flexibility and the overall functionality that they make available to the user. The results of these comparisons are shown in Additional file**1**and Additional file**2**respectively.*
- *We added a new Figure*1*to pictorially illustrate the structure of a CRISPR-Cas site.*
- *In the abstract, we replaced the CRISPR/Cas abbreviation by its full name.*
- *To facilitate the user’s search for a ‘good’ gRNA, we now provide the user with the option to enter an arbitrary target sequence: Off-Spotter will automatically process both strands of the sequence, identify all possible gRNAs for the chosen PAM, and generate separately for each of these gRNAs a list of potential genomic off-targets.*
- *To facilitate the task of sub-selecting among multiple gRNAs we introduced and couple the reported potential off-targets with detailed transcriptomic annotation information from ENSEMBL: by enabling the user to prioritize among gRNAs based on these annotations we make a more informed choice possible. For each analyzed gRNA we also include a histogram showing the number of potential off-targets as a function of the number of mismatches that they have when compared to the gRNA. The output page gives the user the ability to interactively sort the gRNAs and their associated off-targets using a variety of criteria. We stress that it was not our intention to design a new method for ranking gRNAs: the fact that other considerations come into play, some of which are cell-type- and cell-state-specific and outlined in the Discussion and in recent review articles, makes ranking attempts ill-defined in the absence of additional information. Instead of filtering out sites using heuristic criteria, we opted to report all genomic sites whose sequence characteristics make them potential off-targets.*
- *We now make the source code available on our website under*https://cm.jefferson.edu/downloads/

*Lastly, we would like to clarify that Off-Spotter is not a GPU-based solution but an algorithmic one.*

## Additional files

Additional file 1:
**Performance comparisons between Off-Spotter and other similar tools.** Given that the different tools generally allowed different numbers of mismatches when searching for potential off-targets we only used the more commonly available mismatch settings. Additional file 2:
**Qualitative comparison between Off-Spotter and other similar tools.** As can be seen, the various tools offer a variety of features. Generally, the user can select among several PAM strings and define the number of mismatches ahead of time. However, with the exception of Off-Spotter the location and span of the “seed” is hardwired in the algorithm. Also, of all the tested tools, only Off-Spotter, CRISPRseek and Cas-OFFinder report all potential off-targets for a given query gRNA; however, as shown in Additional file 1, Off-Spotter (a software solution) significantly outperforms Cas-OFFinder (a GPU-based system). Also, an important component of gRNA design, namely the annotation of the off-targets sites based on currently available information provided by Off-Spotter is much more extensive compared to all the other tools.

## Abbreviations

CRISPR/Cas: Clustered regularly interspaced short palindromic repeats/CRISPR ASsociated nucleases
gRNA: Guide RNA
PAM: Protospacer adjacent motif
